# Polio in Pakistan: Political, Sociological, and Epidemiological Factors

**DOI:** 10.7759/cureus.3502

**Published:** 2018-10-27

**Authors:** Gabriel E Andrade, Azhar Hussain

**Affiliations:** 1 Psychology, Xavier University School of Medicine, Oranjestad, ABW; 2 Epidemiology and Public Health, Xavier University School of Medicine, Oranjestad, ABW

**Keywords:** polio, pakistan, vaccines, conspiracy theories, epidemiology

## Abstract

Pakistan, Nigeria, and Afghanistan remain the only countries in the world where polio is still a serious threat. This review article addresses the political, sociological, and epidemiological factors behind the failure in eradication. A relatively popular Nigerian conspiracy theory about polio vaccines spread in Pakistan and, as a result, there is some considerable resistance to polio vaccination. Conspiracy theories about vaccines have a long history, and the fact that polio vaccinators were used as spies in order to plan for Bin Laden’s death has made polio vaccination a bigger challenge. Furthermore, vaccination is strongly correlated with literacy and Pakistan is still struggling against illiteracy. However, these obstacles are by no means insurmountable, and a joint effort by the government, policymakers, education department, community mobilizers, and public health professionals can indeed make major improvements.

## Introduction and background

Poliomyelitis is an infectious disease caused by the human enterovirus, poliovirus, which transmits via the fecal-oral route. Its most common symptom is muscle weakness, which results in an inability to move. Although most patients with muscle weakness fully recover, about 2% to 5% of children and 15% to 30% percent of adults die because of respiratory failure [1]. Effective prevention through various rounds of vaccination has ensured the successful eradication of polio in all but three countries: Nigeria, Afghanistan, and Pakistan [2].

Poor sanitary conditions in these countries have made it difficult to fully eradicate the poliovirus [3]. Yet, particularly in the case of Pakistan, sociocultural conditions have made it especially complicated. It is not so much the lack of medical resources that has made polio persistent but, rather, the spread of a particular conspiracy theory that negatively targets vaccinators and non-government organizations (NGOs) working toward the eradication of polio.

This theory claims that vaccination programs seeking to eradicate polio within Pakistan are a scheme created by the United States and Israel to sterilize the Muslim population. The origin of this conspiracy theory has been traced to the 1988 World Health Organization campaign, “Kick Polio Out of Africa.” This was a major effort put forth with the goal of eradicating polio from Africa by the year 2000 [4]. High-profile politicians, such as Nelson Mandela, took special care in promoting this campaign. Through a program of public awareness and door-to-door vaccinations, aid workers were very close to fully eradicating the disease from the entire African continent.

However, in 2003, officials of the regions of Kano, Kaduna, and Zamfara in Nigeria refused to allow vaccine administration in their territories. The rationale was that the polio vaccine may have had an anti-fertility estradiol hormone, human immunodeficiency virus (HIV), and other cancerous agents. In Nigerian public opinion, this view was granted legitimacy because it was defended by a notorious Nigerian physician, Ibrahim Datti Ahmed [5].

This particular physician headed the Supreme Council of Shariah in Nigeria and after the events of September 11, 2001, took an aggressive stand against the West and the United States in particular by publicly proclaiming that polio vaccines were “corrupted and tainted by evildoers from America and their Western allies.” Inasmuch as acquired immunodeficiency syndrome (AIDS) has been a big concern for African countries, Ahmed further added, “We believe that modern-day Hitlers have deliberately adulterated the oral polio vaccines with anti-fertility drugs and viruses, which are known to cause HIV and AIDS” [6]. This kind of rhetoric propelled him to the public view and managed to get him a considerable following.

Although this conspiracy theory was upheld and propagated by Islamist groups in Nigeria, it found the greatest resonance in Pakistan. Public health officials working on vaccination campaigns have been frequently attacked by militant groups, especially in tribal areas controlled by the Taliban [7]. Today, Pakistan remains the country with the highest polio prevalence in the world, and the popularity of this conspiracy theory seems to be the primal causal factor. The eradication of polio in Pakistan, consequently, has to target the eradication of this conspiracy theory.

In order to do so, public health officials and policymakers need to be aware that the popularity of this conspiracy theory in Pakistan does not come out of a vacuum. In fact, despite Pakistani animosity toward the eroding influence of Western culture, the polio vaccine conspiracy theory has Western origins. Mistrust of vaccines has, in fact, a long history. This has also been coupled with a mistrust of physicians and healthcare professionals, as a result of some infamous cases of gross violations of ethical guidelines [8]. Suspicions regarding population control and covert anti-fertility programs, which are a significant part of this theory, also go back in time.

Yet, despite the Western origins of many elements of this conspiracy theory, Pakistan’s complex place in the geopolitical stage after the attacks of September 11, 2001, and its uneasy relationship with the United States ever since, have made it a fertile sociological ground for conspiracy mongering. In this article, we will review some of the historical and sociological background to the polio vaccine conspiracy theory in Pakistan.

## Review

The history of conspiracy theories regarding vaccinations goes back to the very first vaccine. The word vaccine comes from the Latin *vaccinae*, meaning “of the cow.” The word was originally created by Edward Jenner, an 18th Century English country gentleman. At the time, smallpox was rampant throughout Europe. In fact, smallpox was only officially eradicated in 1980 but, by far, it has been the disease that has killed the greatest number of human beings [9].

Jenner had many scientific interests; however, given the frequent devastation of smallpox, Jenner sought to explore ways of preventing the disease. His social interactions with English farmers and cattle herders led him to discover that those people who were in frequent contact with cows had a lower propensity to suffer from smallpox. He was aware of the folk wisdom that claims that cowpox (a disease that causes blisters on cows’ udders) can prevent against smallpox in humans. Jenner thus proceeded to expose farmers who had suffered cowpox to the smallpox virus. None were infected. He then deliberately infected a boy with cowpox and then exposed him to smallpox. The boy did not get sick; thus, the first vaccine was born [10].

Needless to say, Jenner’s contribution to the history of medicine was monumental, though it is very doubtful that it complied with ethical standards [11]. Jenner carried out the experiment on a healthy young boy, without being certain in advance what the results would be. Clearly, the boy did not have the capacity to give informed consent, a cardinal principle in medical ethics to be an experimental subject. Had the experiment gone bad, great damage would have been done, as the boy was healthy prior to the experiment.

Perhaps this troublesome moral aspect of Jenner’s achievement was an important factor in the public’s reaction to his proposed method of curing smallpox. The fact that an apparently risky procedure was done on a healthy boy aroused many moral suspicions, even if, indeed, time proved that Jenner was right.

Vaccines are, for the most part, administered during childhood. At that age, individuals cannot truly give consent, which makes vaccines morally suspicious among some people. To a certain extent, this may be the case with the mistrust of polio vaccination in Pakistan. Furthermore, Jenner’s procedure seemed very counterintuitive at the time. How could exposure to a similar virus actually prevent a disease as deadly as smallpox? We now know how vaccines work. They stimulate the immune system [12]. During Jenner’s time, this was not fully understood; thus, it was comprehensible that people exhibited distrust of a cure that does not seem to be altogether different from the disease itself.

Indeed, Jenner’s innovation was not immediately well-received. Certain religious groups argued that men should not alter God’s plan. The fact that the cure for smallpox came from ill cows disgusted many. There were further concerns about what unforeseen consequences such a medical procedure could also bring forth.

In the scientific community, Jenner’s innovation was quickly accepted, and public health officials soon realized that the only effective way of controlling smallpox was by imposing mandatory vaccination, as in the Compulsory Vaccination Acts of the 1850s passed by the British Parliament [13]. Being inspired by the strong British tradition of individual autonomy and liberty, the laws were eventually opposed by groups who sought to resist mandatory vaccination. These vaccination groups soon spread beyond Great Britain in the late 19th Century.

The original anti-vaccination campaign was derived from a basic political argument that claims that individuals are entitled to control of their own body and, thus, it is oppressive to impose mandatory vaccination. However, as too often has been the case, anti-vaccination campaigns have been far more driven by irrational and emotional appeals. Even during Jenner’s time, his critics claimed that recipients of the smallpox vaccine could grow horns, implying a falsified notion that vaccinated individuals would become cow-like as a result of the cowpox virus in their system [14]. Ultimately, of course, the smallpox vaccination was successful, and the disease was ultimately eradicated in the late 1970s [15]. However, since then, the same anti-vaccination arguments have been constructed against vaccines of other diseases.

The most recent anti-vaccination campaign relates to the measles, mumps, and rubella (MMR) vaccine and its alleged link to autism [16]. In 1998, Dr. Andrew Wakefield, along with 12 other authors, published an article reporting the case studies of 12 children in the United Kingdom [17]. These children were diagnosed with certain developmental disorders, autism being the most common. The children’s parents reported that they began to notice the symptoms of developmental disorders in their children soon after the administration of the MMR vaccine.

Wakefield then proceeded to perform intestinal biopsies on the subjects, and the results came out positive for inflammation. According to Wakefield et al., vaccines were causing this intestinal inflammation. The study had some considerable methodological flaws. Some of the patients’ parents reported that their children had symptoms of autism before symptoms of bowel disease. This fact does not cohere well with Wakefield’s theory. If autism is a result of intestinal inflammation, it would be normal to expect that symptoms of the latter would actually appear first; yet, that was not the case. In fact, video footage of autistic children’s first birthday parties has been compared to footage of non-autistic children’s first birthday parties, and researchers are able to predict, with a high degree of accuracy, which children turned out to be autistic and which did not. This is strong evidence that symptoms of autism chronologically antecede the administration of vaccines, which, in turn, suggests that vaccines do not cause autism [18].

It later became public that there were very serious ethical issues with Wakefield’s original study. In 2004, 10 of the authors of Wakefield’s original article retracted. According to some journalistic investigations by Brian Deer, Wakefield received financial incentives from lawyers of parents of autistic children, who had the intention of taking legal action against the companies that manufactured the MMR vaccine. Wakefield never undisclosed this conflict of interest. Furthermore, Wakefield had the intention of registering a patent for a vaccine that, if launched in the market, would have competed with the conventional MMR vaccine [19].

Despite strenuous debunking of both the ethics and methodology of Wakefield’s study, his claims managed to resonate with a growing sector in the United States and the United Kingdom. As a result of Wakefield’s infamous study, the vaccination rates of MMR have dropped significantly, and expectedly, some occasional outbreaks of measles, mumps, and rubella have been reported [20].

Wakefield’s original article had little conspiracy mongering, but as soon as his claims became popular in the media, conspiracy theorists added to his original allegations by claiming that “Big Pharma” was behind the MMR vaccine and that the rise in autism prevalence was actually a direct consequence of the pharmaceutical companies’ profit [21].

Tellingly, only five years after Wakefield’s article, the polio vaccine conspiracy theory was first formulated in Nigeria. There is no formal data to support the hypothesis that Wakefield’s article was ultimately a causal factor, but we may conjecture that, indeed, Wakefield’s article contributed to the environment of mistrust in Nigeria and Pakistan regarding vaccines in general.

Population control conspiracy theories

Prior to industrialization, fertility was highly valued universally and there was little concern with population control. With insufficient levels of technological advancement, manpower was highly valued for the production of wealth. However, as the Industrial Revolution took shape in Western Europe during the late 18th Century, it was accompanied by the so-called “demographic transition.” Cities became more crowded, and as technologies began to replace manpower, overpopulation became a concern [22].

This was explicitly addressed by Robert Malthus in his classical 1798 book, An Essay on the Principle of Population. Malthus argues that food resources grow at an arithmetic rate, whereas the population grows at an exponential rate. This would eventually cause a mismatch, and populations would be pressured to reduce their sizes through war, disease, and famine. Malthus disapproved of contraceptive methods but insisted on the need for abstinence for purposes of population control. He also opposed laws and social programs that would provide some assistance to the poor, as he thought such initiatives encouraged population growth [23].

Malthus’ opposition to welfare programs was enthusiastically taken by adherents of social Darwinism in the later 19th Century [24]. Given their ruthlessness and zeal in hoping to rid society of its undesirable population, this paved the way for conspiracy theorists to claim that there is a global effort to control population size through covert malicious means.

The Club of Rome, a high-profile intellectual organization created in the 1970s, took a neo-Malthusian approach to address many global issues and published a widely read report, The Limits to Growth. The book warned about potential problems if growth was not controlled [25]. Yet, conspiracy theorists soon claimed something far more sinister was going on behind closed doors. In fact, as the allegation went, the Club of Rome was targeting impoverished nations to reduce their population size [26]. This was coupled with the claim that environmentalist groups, of which some members of the British Crown are fond of, most notably Prince Phillip, are also actively seeking to control population growth and even actively make the human species go extinct (as in the Voluntary Human Extinction Movement) in order to protect nature [27]. 

 As a result of all these theories, a common trope of conspiracy mongering has been the so-called “New World Order,” an imagined project headed by the Illuminati, whose goal is to establish global rule through puppet governments, by constantly keeping in check population size, especially in Third World countries. Conspiracy theorists have traditionally believed this project is already underway, through things such as water fluoridation, genetically modified food, and airplane contrails (erroneously believed by conspiracy theorists to be poisonous “chemtrails”).

Vaccines play an important role in this conspiracy theory. According to influential conspiracy theorist Leonard Horowitz (1997), the medical industry is actively seeking to control population size through the continuous manufacture of new viruses [28]. AIDS and Ebola, as this theory claims, were deliberately inoculated in human communities in order to decrease population size. Vaccines, especially the polio vaccine, also have population control as a goal.

Horowitz claims that vaccines control population size by deliberately killing people. Yet, that is not exactly the same claim made in Pakistan. The claim made by conspiracy theorists in Pakistan, following the original Nigerian version, is that vaccines only contribute to infertility. A common documented trend in the formation of conspiracy theories is how bits and pieces are amalgamated into a unified whole, even though originally, they may be have been unrelated to each other [28-29].

Pakistan’s fertile ground for conspiracy theories

The distrust of vaccines and conspiracy theories regarding population control have been mostly a phenomenon of Western origin. Yet, sociology has documented that conspiracy-mongering thrives more in regions with specific sociological and cultural circumstances [30]. Unfortunately, Pakistan is one of those countries in which such circumstances have flourished, and this has made it all too easy for this nation to import and adapt these harmful conspiracy theories.

The most important social predictor of belief in conspiracy theories is perceived powerlessness and a sense of lack of control [31]. This is true both at a psychological and a sociological level. Nations that have a historical sense of being cheated, and anxiety over the idea that their fate is controlled by outside forces, have a higher probability of developing conspiracy theories.

Pakistan itself was born out of the concern that, in a unified India, Muslims would be persecuted by Hindus. Pakistan’s founder, Muhammad Ali Jinnah may or may not have been right in his warnings about a unified India, but the fact remains that, from the very moment of its birth, Pakistan, as a nation, was immersed in paranoia over its hostile and more powerful neighbor. This sense of suspicion was reinforced when India annexed Muslim-majority Kashmir upon partition, and in four consecutive wars, defeated Pakistan. These experiences added to a very unstable political situation that led to military coups in 1953, 1958, 1977, and 1999 [32].

Pakistan’s place in the geopolitical scenery and uneasy relationship to the West (especially the United States) has further contributed to the collective feeling of suspicion and lack of control of its own affairs. During the Cold War, as India’s ties to the Soviet bloc seemed to grow closer, Pakistan received considerable military and economic support from the United States. This was further intensified when, under Zia Ul Haq’s regime, Pakistan offered substantial support to Afghanistan’s mujahideen in their armed resistance to the Soviet invasion [33]. The United States found in Pakistan an important ally to counter Soviet expansionism.

The attacks on New York City on September 11, 2001, changed the situation dramatically. The Soviet Union no longer existed and, now, Pakistan was scrutinized by the United States as a possible collaborator with terrorist militant groups. Under Pervez Musharraf’s regime, Pakistan pledged alliance to the United States in the so-called “war on terror,” and received massive financial and military aid. Unlike the prior collaboration during the Afghan-Soviet war, this time, there was wide suspicion in the American government that Pakistani authorities were actually harboring militants [7,33-34].

The United States then began an aggressive program of drone attacks violating Pakistani sovereignty and disregarding civilian deaths. This, in turn, massively contributed to suspicion of American integrity amongst the Pakistani population. The American government continuously claimed that Osama Bin Laden was hiding in Pakistan, and in 2011, again violating Pakistani sovereignty, conducted a military raid that ended his life in the Pakistani city of Abbottabad [7,34].

Upon Bin Laden’s death, various conspiracy theories began to circulate in Pakistan. Some groups claimed that Bin Laden died shortly after the attacks of 2001, as he had been suffering from Marfan syndrome; the United States pretended that he was still alive, as an excuse to launch the “War on Terror.” Other groups claimed that Bin Laden was killed somewhere else, as he was never in Pakistan in the first place, but the United States made up the story as an excuse to exert military control over Pakistan. Other groups even claim that Bin Laden is still alive. The fact that Abbottabad hosts an important military academy increased the suspicion that something was not right in the official version of Bin Laden’s death, as it could hardly have taken place without Pakistani military personnel noticing [34].

These conspiracy theories have no empirical support whatsoever. Yet, regarding Bin Laden’s death, there is one conspiracy theory that has turned to be true. It was reported that, as part of planning for the military operation that killed Bin Laden, spies posing as polio vaccinators got close to Bin Laden’s home and collected information about his whereabouts. This claim has been sufficiently confirmed by multiple independent sources [35], including Dr. Shakil Afridi, who led the espionage operation [36].

This military tactic has been widely criticized by medical ethicists, both for its deceptive nature and for the potential harm that it may cause in the future [37]. Indeed, the harm is already evident. The CIA’s use of spies posing as vaccinators has given some legitimacy to the original conspiracy theory that polio vaccines are part of an evil plot. Of course, polio vaccination campaigns have not been used by Western powers to make Pakistani children infertile, but they were used by a Western power to kill a militant, in violation of Pakistani sovereignty. In the minds of lesser-educated Pakistanis (the ones who are most vulnerable to polio), it is fairly easy to make the leap and conclude that, all along, polio vaccinations have been done with malicious intentions, and they can only lead to bad results [8].

Description of the epidemiological situation of polio in Pakistan

Despite the fact that there are only three countries, including Pakistan, which are still affected by the poliovirus, Pakistan is making some progress toward the eradication of polio. In Baluchistan, a province in Pakistan, a circulating vaccine-derived poliovirus type 2 (cVDPV2) has been collected and verified from the environmental samples [38]. Pakistan is also the hub of endemic transmission of wild poliovirus type 1 (WPV1). According to a World Health Organization (WHO) report, until December 19, 2016, only 21 cases affected by WPV1 were reported. While comparing the previous number of WPV1 affected cases, this number of 21 in a year was the lowest.

It is becoming difficult to eradicate polio from Pakistan, as the roots of poliovirus are found and confirmed in almost all cities, even metropolitan cities like Karachi, Peshawar, and Quetta. These metropolitan cities are capable enough to transmit the polio virus to other cities and to the remote areas in Pakistan.

To understand the prevalence and reasons behind the poliovirus, it is also necessary to analyze the literacy rate and epidemiological conditions in the area. In Pakistan, there are places like Sindh and Balochistan where the literacy rate is even below 20% [35,37]. This literacy rate is not important in terms of family and parents being educated and aware about the poliovirus and immunization, but it is linked with the awareness and education status of the health workers of those areas. A survey was conducted in the Quetta and Peshawar regions, which found that health workers who are more qualified and educated are more updated and aware about the polio program and these people are more dedicated and committed towards the eradication of polio from Pakistan [39]. It is obvious by this fact that places, where the health care workers are more qualified, can motivate and aware people in their territory regarding the polio program and steps required to eradicate this from Pakistan.

In Pakistan, three high polio transmission zones (HPTZ) have been identified so that the corrective and preventive actions can be planned in these regions. These identified places are Khyber Pakhtunkhwa (KPK) region, Federally Administered Tribal Areas (FATA), and the third identified zone is the metropolitan city Karachi. In all these three polio transmission zones, there are total 33 districts, which are identified as “highly endangered districts.”

Alternatives, hopes, and proposal for public policy

With declining rate and number of cases detected with poliovirus, government, and other stakeholders like WHO and United Nations (UN) organizations are more inclined towards the eradication of polio, not only from Pakistan but globally. Comparing the last few figures of reported cases, poliovirus-affected individuals were 282 in the year 2014 and declined tremendously to 49 cases in 2015 [40]. After the deaths and attack on health care workers, it is time to reassure the health care workers who are responsible to train and aware the people from the community on the polio program and steps required to eradicate this disease from the society. Security to health care workers and community engagement could help in training and awareness program for the communities [41].

For the community engagement and acceptance for any public health program, one of the contributing factors is engaging someone from the local community who is easily welcomed and accepted by the community. For polio eradication program, one of the key recommendations is engaging and hiring local women who represent their society and they are well-welcomed in the society. They can motivate and aware of the local community more easily as compared with someone from outside the community [35,40]. These local women will be easily trusted by the parents when it comes to vaccination.

One of the studies was conducted on the behavior and attitude of healthcare workers towards poliovirus and its effect on children, and the result was quite surprising. In this study, it was found that the healthcare workers themselves rate poliovirus not a challenge for children in Pakistan [42]. In this study, the researcher was astonished when he realized that only 1.6 percent of the health workers take poliomyelitis as one of the challenging health issues. It is important for the policymakers to first have an awareness program of healthcare workers so that they understand how big this issue is. There should be a collaborative approach where healthcare workers from Pakistan interact and talk with healthcare workers of other countries where polio is not prevalent now. This approach will help the healthcare workers about their importance, roles, and responsibilities in eradicating polio from Pakistan.

## Conclusions

Considering the challenges in Pakistan in regards to the expanding spread of poliovirus, a collaborative effort should be created by the government, policymakers, education department, community mobilizers, and public health professionals to understand the severity of polio and how to begin the journey towards its eradication from Pakistan. The country faces opposition towards the vaccination as a result of a combination of the historical, sociological, and political perspectives cultivated in the region. However, tackling the misunderstandings that the Pakistani population has towards vaccines will allow for more successful attempts to eradicate the poliovirus from the country.
